# ALDH Expression Characterizes G1-Phase Proliferating Beta Cells during Pregnancy

**DOI:** 10.1371/journal.pone.0096204

**Published:** 2014-05-02

**Authors:** Lijuan Zhang, Lin Wang, Xiaoliang Liu, Dongming Zheng, Sishi Liu, Caixia Liu

**Affiliations:** The Department of Gynecology and Obstetrics, Shengjing hospital affiliated to China Medical University, Shenyang, China; Bascom Palmer Eye Institute, University of Miami School of Medicine, United States of America

## Abstract

High levels of aldehyde dehydrogenase (ALDH) activity have been detected in various progenitor and stem cells. Thus, Aldefluor fluorescence, which represents precisely the ALDH activity, has been widely used for the identification, evaluation, and isolation of stem and progenitor cells. Recently, ALDH activity was detected in embryonic and adult mouse pancreas, specifically in adult centroacinar and terminal duct cells supposed to harbor endocrine and exocrine progenitor cells in the adult pancreas. Nevertheless, ALDH activity and aldeflour fluorescence have not been examined in beta cells. Here, we report a dynamic increase in the number of aldeflour+ beta cells during pregnancy. Interestingly, nearly all these aldeflour+ beta cells are positive for Ki-67, suggesting that they are in an active cell cycle (G1, S and M phases). To determine precisely at which phase beta cells activate ALDH activity and thus become aldeflour+, we co-stained insulin with additional proliferation markers, phosphohistone3 (PHH3, a marker for M-phase proliferating cells) and Bromodeoxyuridine (BrdU, a marker for S-phase proliferating cells). Our data show little aldeflour+ beta cells that were positive for either PHH3, or BrdU, suggesting that beta cells activate ALDH and become Aldefluor+ when they enter G1-phase of active cell cycle, but may downregulate ALDH when they leave G1-phase and enter S phase. Our data thus reveal a potential change in ALDH activity of proliferating beta cells during pregnancy, which provides a novel method for isolation and analysis of proliferating beta cells. Moreover, our data also suggest that caution needs to be taken on interpretation of Aldefluor lineage-tracing data in pancreas.

## Introduction

Diabetes is a metabolic disease resulting from dysfunction and/or loss of pancreatic insulin-secreting beta cells, and is characterized by chronic hyperglycemia [1]. Since increase in functional beta cell mass may be a fundamental cure for diabetes, great efforts have been made to search for new sources of beta cells. Previous studies have suggested that cell replication is the predominant mechanism for postnatal beta cell growth [2]–[6]. There were also reports of evidence for beta cell neogenesis [7], [8], which were not supported by follow-up studies [9]–[12]. Researchers have focused on the study on the mechanism by which beta cells is stimulated to enter an active cell cycle, since the turnover of adult beta cells is typically extremely slow [13]–[17].

Postnatal beta cell growth occurs in some situations, which are used as models for studying the molecular basis of beta cell replication. Among these situations, pregnancy appears to be the strongest physiological stimulus for postnatal beta cell growth [18]–[22]. However, most previous studies have been performed using partial pancreatectomy model [23].

Increased activity of aldehyde dehydrogenase (ALDH), a detoxifying enzyme responsible for the oxidation of intracellular aldehydes [24], [25], has been detected in some stem/progenitor cells. For example, high ALDH activity has been found in murine and human hematopoietic and neural stem and progenitor cells [26]–[29]. Recently, ALDH activity was detected in embryonic and adult mouse pancreas, specifically in adult centroacinar cells and terminal duct cells supposed to harbor endocrine and exocrine progenitor cells in the adult pancreas [30]. Nevertheless, ALDH activity and aldeflour fluorescence (representing ALDH activity) have yet been examined in beta cells.

Here, we report a dynamic increase in the number of aldeflour+ beta cells during pregnancy. Interestingly, nearly all these aldeflour+ beta cells are positive for Ki-67, suggesting that they are in an active cell cycle (G1, S and M phases). To determine precisely at which phase beta cells activate ALDH activity and thus become aldeflour+, we co-stained insulin with additional proliferation markers, phosphohistone3 (PHH3, a marker for M-phase proliferating cells) and Bromodeoxyuridine (BrdU, a marker for S-phase proliferating cells). Our data show little aldeflour+ beta cells that were positive for either PHH3, or BrdU, suggesting that beta cells activate ALDH and become Aldefluor+ when they enter G1-phase of active cell cycle, but may downregulate ALDH when they leave G1-phase and enter S phase. Our data thus reveal a potential change in ALDH activity of proliferating beta cells during pregnancy, which provides a novel method for isolation and analysis of proliferating beta cells. Moreover, our data also suggest that caution needs to be taken on interpretation of Aldefluor lineage-tracing data in pancreas.

## Materials and Methods

### Mouse handling

All mouse experiments were approved by the Institutional Animal Care and Use Committee at Shengjing Hospital of China Medical University (Animal Welfare Assurance). Surgeries were performed under ketamine/xylazine anesthesia, according the Principles of Laboratory Care, supervised by a qualified veterinarian. All efforts were made to minimize pain and suffering. Female Balb/C mice of 12 weeks of age were used in the current study. Four mice were analyzed in each experimental condition. 50 mg/kg Bromodeoxyuridine (BrdU, Sigma, China) was intraperitoneally injected two hours before sacrifice for labeling proliferating beta cells.

### Bone marrow and islet isolation and analysis of Aldefluor+ islet cells by flow cytometry

Bone marrow cells were isolated as has been previously described [31], [32].The mouse pancreas was perfused with 30 mg/dl collagenase (Sigma, China) from the common bile duct, and then incubated in a 37°C shaker for 30 minutes at a speed of 200 times per minute. After centrifugation, the pellet was resuspended in Histopaque (Sigma) of a gravity of 1.12 for a subsequent gradient centrifugation at 1200 rpm for 20 minutes. The suspension fraction was used for serial islet hand-pickings. Islet purity was assured by absence of exocrine cell markers Sox9 and Amylase. Purified islets were further digested with 10 µg/ml trypsin (Sigma) for 25 minutes to prepare single cell fraction for flow cytometry. The Aldefluor Kit (StemCell Technologies, China) was applied according to the manufacturer's instructions, to identify high ALDH enzymatic activity. Flow cytometry was performed using a FACSAria (Becton Dickinson) flow cytometer. Immunostaining on cytospun cells were performed as has been previously described [33].

### Immunohistochemistry

Mouse pancreas were dissected out and fixed with 4% Paraformaldehyde for 10 hours, and then cyro-protected in 25% sucrose overnight. Samples were then sectioned in 6 µM and immunostained with guinea pig polyclonal insulin, rat polyclonal BrdU and Ki-67, rabbit polyclonal ALDH (ab23375), phosphohistone 3 (PHH3) and Ki-67 antibodies (all purchased from Abcam). BrdU staining needs antigen retrieval, which was performed by incubation of the slides with 1 mol/l HCl at room temperature for 45 minutes. Secondary antibodies were Cy3- and Cy2- conjugated antibodies for corresponding species (Jackson Labs, USA).

### Quantification of beta cell replication

Four mice were measured in each experimental condition. Insulin staining was used to identify beta cells. The quantification of ALDH+ cells in all Ki-67+ beta cells, or ALDH+ cells in all BrdU+ beta cells, or ALDH+ cells in all PHH3+ beta cells, was based on 5 sections that were 100 µm apart from each other. 1500∼3000 beta cells were counted in each animal (Table S1).

### Statistics

All values are depicted as mean ± standard deviation from 4 individuals and are considered significant if p<0.05. All data were statistically analyzed using one-way ANOVA with a Bonferoni correction.

## Results

### Aldefluor+ cells are detected in the islets of pregnant mice

**Figure 1:**
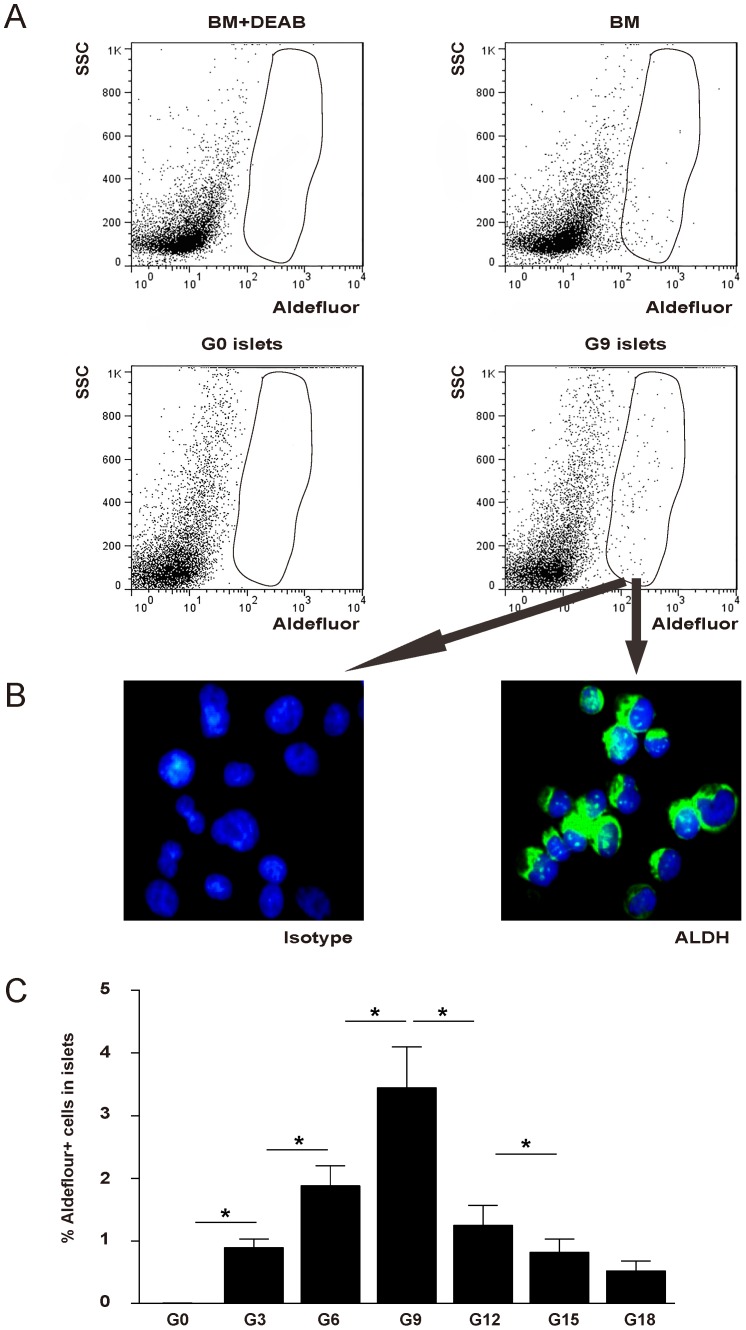
Aldefluor+ cells are detected in the islets of pregnant mice. (A) Representative flow cytometry analysis of aldefluor fluorescence in the bone marrow cells (+/− treatment with a specific ALDH inhibitor DEAB) and isolated islets from pregnant mice 9 days after pregnancy (G9), compared with non-pregnant mice (G0). While no aldefluor+ cells were detected in G0 islets, aldefluor+ cells (circled) were readily detected in the islets from G9 islets. (B) Isolated G9 aldeflouor+ cells were immunostained positive for ALDH. (C) Quantification of aldefluor+ cells in the islet fraction from pregnant mice at 3, 6, 9, 12, 15 and 18 days after pregnancy (G3, G6, G9, G12, G15 and G18, respectively). These data show that in the mouse pancreas, islet cells upregulate ALDH activity during pregnancy. SSC: side-light scatter. *: p<0.05.

Aldefluor fluorescence represents precisely the ALDH activity, and has been widely used for the identification, evaluation, and isolation of stem and progenitor cells. Therefore, we first examined the aldefluor fluorescence in the isolated islets from pregnant mice at 3, 6, 9, 12, 15 and 18 days after pregnancy (G3, G6, G9, G12, G15 and G18, respectively), compared with non-pregnant mice (G0) of the same age (12 weeks of age) at the same period. Bone marrow cells were used as a positive control, while bone marrow cells pre-treated with 1.6 mM diethylaminobenzaldehyde (DEAB), a specific ALDH inhibitor, was used as a negative control (Fig. 1A). Isolated aldeflouor+ cells were immunostained positive for ALDH (Fig. 1B). While no aldefluor+ cells were detected in G0 islets, aldefluor+ cells were readily detected in the islets from the mice at different times in the pregnancy (Fig. 1C: G3: 0.89±0.14% in total islet cells, G6: 1.88±0.32%, G9: 3.45±0.65%, G12: 1.25±0.32%, G15: 0.82±0.21%, G18: 0.52±0.16%). To our knowledge, our study is the first to show that mouse islet cells upregulate ALDH activity during pregnancy.

### ALDH+ cells in the islets are predominantly beta cells

**Figure 2:**
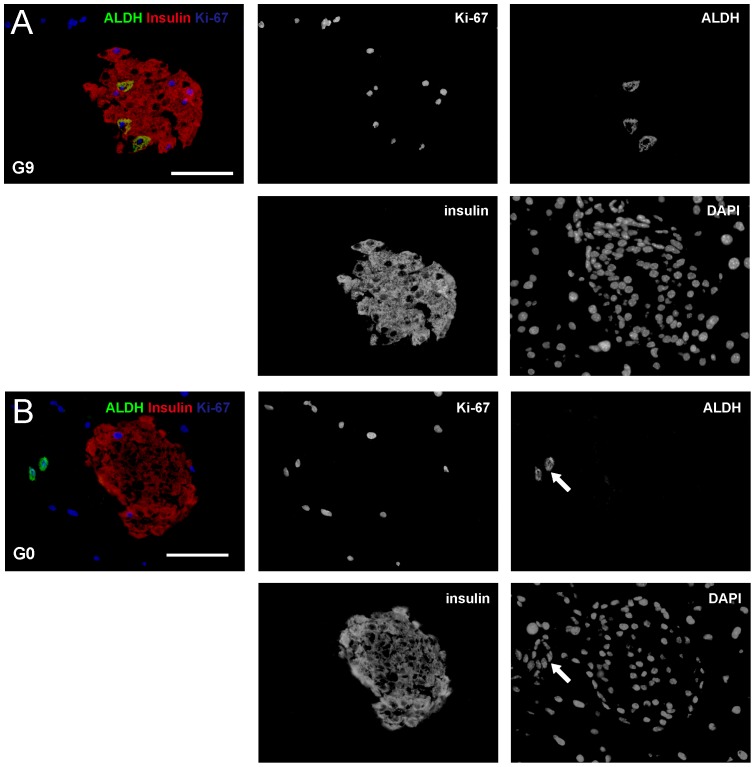
ALDH+ cells in the islets are predominantly beta cells in active cell cycle. (A–B) Representative triple staining for ALDH (in green), insulin (in red) and Ki-67 (in blue), together with nuclei staining with DAPI are shown in G9 (A) and G0 (B) pancreas. Each channel was shown independently. A merged image for ALDH, insulin and Ki-67 was also shown. The result suggests that aldefluor+ cells in the islets are predominantly proliferating beta cells. Arrows point to ALDH+ terminal cells. Scale bar is 50 µm.

To confirm the findings from the analysis on aldefluor fluorescence, and to identify which cell types in the islets from pregnant mice have increased ALDH activity, we performed immunostaining for ALDH and insulin on G9 mouse pancreas, since our data show the highest aldefluor fluorescence in G9 islets. G0 mouse pancreas was used as a control. We found that 4.5±0.7% of beta cells (identified by insulin staining) were positive for ALDH (Fig. 2A). In control G0 pancreas, although ALDH positivity was readily detected in some centroacinar and terminal ductal cells, we did not find any ALDH+ cells in the islets (Fig. 2B). These data suggest that aldefluor+ cells in the islets are predominantly beta cells.

### ALDH+ beta cells are predominantly in active cell cycle

We then aimed to examine the difference between ALDH+ beta cells and ALDH- beta cells. We first tested whether ALDH activity may relate to cell cycle activity, since it is well-known that beta cells substantially increase proliferation during pregnancy to increase beta cell mass in response to metabolic demand. Thus, we co-stained ALDH, insulin with a proliferation maker Ki-67, which labels all cells in G1, S and M phases of an active cell cycle. We found that 99.7±1.3% of ALDH+ beta cells were positive for Ki-67. Moreover, in all Ki-67+ beta cells, 32.5±2.2% were positive for ALDH (Fig. 2A-B). These data suggest that aldefluor+ cells in the islets are predominantly proliferating beta cells.

### ALDH+ beta cells are predominantly not M-phase proliferating cells

**Figure 3:**
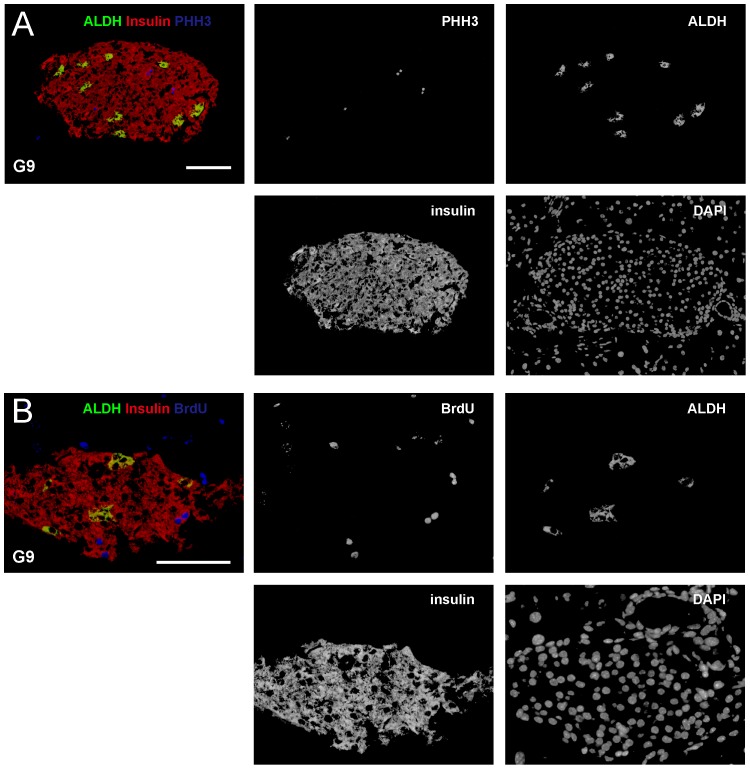
ALDH+ beta cells are not in M or S phase. (A–B) To determine precisely at which phase of a cell cycle, beta cells activate ALDH activity and thus become ALDH+, we co-stained ALDH (in green), insulin (in red) with PHH3 (in blue) and BrdU (in blue). BrdU was given to the mice two hours prior to sacrifice. (A) Representative images show essentially no PHH+ALDH+ beta cells, suggesting that beta cells lose ALDH activity when they enter M phase of a cell cycle. (B) Representative images show essentially no BrdU+ALDH+ beta cells, suggesting that beta cells lose ALDH activity when they enter S phase of a cell cycle. Scale bar is 50 µm.

To determine precisely at which phase of a cell cycle, beta cells activate ALDH activity and thus become aldeflour-positive, we co-stained ALDH, insulin with additional proliferation markers, phosphohistone 3 (PHH3, a marker for M-phase proliferating cells) and Bromodeoxyuridine (BrdU, a marker for S-phase proliferating cells). BrdU was given to the mice two hours prior to sacrifice, since 2 hours is not long enough for a beta cell in late S-phase to pass M phase, split, then pass G0 phase and enter G1 phase again. Only a cell that does so within 2 hours in the current setting may affect our interpretation of data. This is very unlikely to occur, according to previous work that has demonstrated long G0 phase for beta cells [34]–[36]. We did not detect PHH+ALDH+ beta cells (less than 0.5%) (Fig. 3A), suggesting that beta cells may lose ALDH activity when they ender M phase of a cell cycle.

### ALDH+ beta cells are predominantly not S-phase proliferating cells

**Figure 4:**
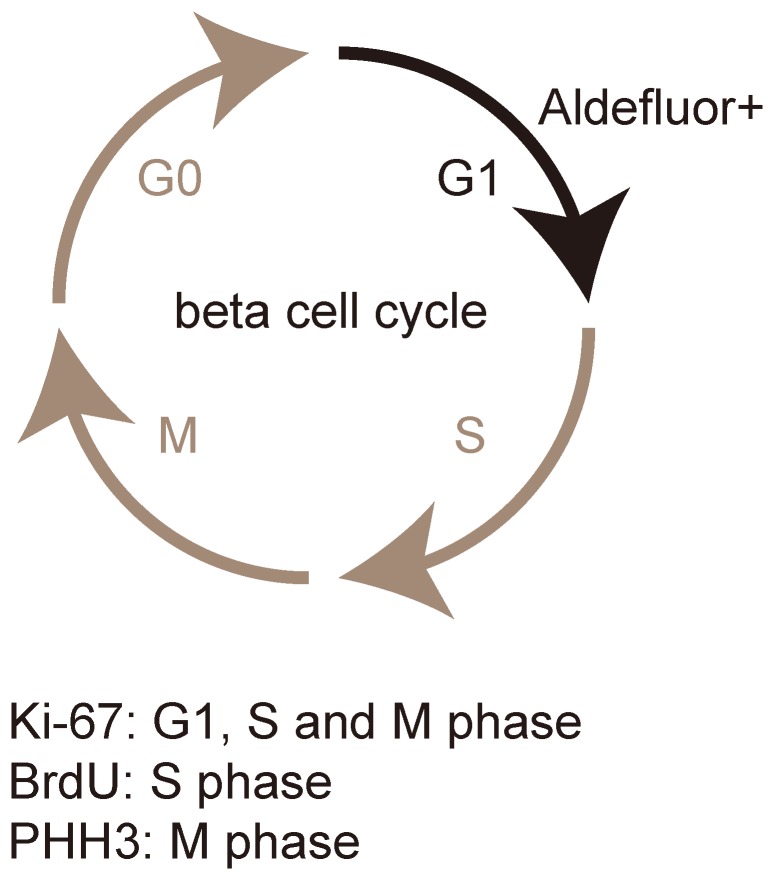
Beta cells increase ALDH activity in G1 phase during proliferation. Our finding was summarized and illustrated. Beta cells activate ALDH and become aldefluor+ when they enter G1-phase of an active cell cycle, but may downregulate ALDH and become Aldefluor- when they leave G1-phase and enter S phase.

Similarly, we detect very few BrdU+ALDH+ beta cells (1.25±0.2%) (Fig. 3B), suggesting that beta cells essentially loss ALDH activity when they ender S phase of a cell cycle. Taken together, our data suggest that beta cells activate ALDH and become Aldefluor+ when they enter G1-phase of active cell cycle, but may downregulate ALDH when they leave G1-phase and enter S phase. These findings were then summarized and illustrated in Fig. 4.

## Discussion

ALDH activity is one identifying marker for normal and malignant stem cells [37], [38]. Assays for measuring ALDH activities include Western blot, RT-PCR, spectrophotometric assay for enzyme activity, and immunohistochemistry. Flow cytometry-based aldefluor staining is a relatively new method for measuring ALDH activity in viable cells and has made it feasible to study ALDH expression in murine and human cells. Recently, aldefluor was used to sort the ALDH+ population of central acinar/terminal duct cells from peripheral acinar duct units of adult mice [30]. These cells expressed early embryonic pancreas markers and formed spherical pancreatospheres. The derived endocrine cells showed glucose regulated insulin secretion. On transplantation into mouse embryos, ALDH+ cells were found to contribute to both exocrine and endocrine lineages in the developing pancreas [30]. This study supported the concept that beta cell neogenesis may occur in adults from the ALDH+ central acinar/terminal duct cells, challenging present general belief of a nearly exclusive contribution of beta cell proliferation to postnatal beta cell growth [2]–[6], and was not supported by the result from another independent study [39].

In the current study, we detected a dynamic increase in the number of aldeflour+/ALDH+ beta cells during pregnancy. We further show that nearly all these ALDH+ beta cells are also positive for Ki-67, suggesting that ALDH+ beta cells are mainly in an active cell cycle (G1, S and M phases). Moreover, co-staining of insulin and ALDH with additional proliferation markers, PHH3 and BrdU, suggest that beta cells activate ALDH and become aldefluor+ when they enter G1-phase of an active cell cycle, but may downregulate ALDH and become Aldefluor- when they leave G1-phase and enter S phase. Since G2-phase is very brief, we did not specifically analyze G2-phase. However, our data do not support a greater ALDH activity of beta cells in G2-phase.

To our knowledge, our study is the first to show that ALDH activity can be detected in proliferating beta cells. The existence of adult beta cell neogenesis is very controversial. Here we provided strong evidence to argue against using ALDH activity or aldefluor fluorescence as a marker for beta cell progenitor/stem cells in the adult pancreas, and suggest that caution needs to be taken during interpretation of Aldefluor lineage-tracing experiments.

Moreover, since we precisely defined the activation window of ALDH in beta cells, our finding may lead to a novel method for isolation and analysis of proliferating beta cells, and specifically proliferating beta cells in G1 phase.

## Supporting Information

Table S1 Quantification of ALDH, Ki-67-positive beta cells.
(DOCX)
