# Supplementary material for: ALDH Expression Characterizes G1-Phase Proliferating Beta Cells during Pregnancy
Source: PLoS One. 2014 May 2;9(5):e96204. doi: 10.1371/journal.pone.0096204 (PMC4008573; doi:10.1371/journal.pone.0096204)
Supplement: Table S1 — Quantification of ALDH, Ki-67-positive beta cells. (DOCX) [file pone.0096204.s001.docx]

Table S1: quantification of ALDH, Ki-67-positive beta cells

| Mouse ID | beta cells counted | Ki-67+ cells | ALDH+Ki-67+ cells | ALDH+Ki-67- cells | %ALDH+beta cells | %ALDH+ cells of Ki-67+ cells |
| --- | --- | --- | --- | --- | --- | --- |
| G0-1 | 1887 | 8 | 2 | 0 | 0.11 | 25.00 |
| G0-2 | 1679 | 7 | 2 | 0 | 0.12 | 28.57 |
| G0-3 | 1909 | 6 | 1 | 0 | 0.05 | 16.67 |
| G0-4 | 1989 | 13 | 3 | 0 | 0.15 | 23.08 |
| Mean |  |  |  |  |  |  |
| SD |  |  |  |  |  |  |
| G9-1 | 2476 | 345 | 102 | 0 | 4.12 | 29.57 |
| G9-2 | 2776 | 289 | 101 | 0 | 3.64 | 34.95 |
| G9-3 | 2045 | 314 | 104 | 1 | 5.09 | 33.12 |
| G9-4 | 1987 | 309 | 100 | 0 | 5.03 | 32.36 |
| Mean |  |  |  |  | 4.47 | 32.50 |
| SD |  |  |  |  | 0.71 | 2.24 |
| Mouse ID | beta cells counted | BrdU+ cells | ALDH+BrdU+ cells | ALDH+BrdU- cells | %ALDH+beta cells | %ALDH+ cells of BrdU+ cells |
| G9-1 | 1978 | 102 | 1 | 101 |  | 0.98 |
| G9-2 | 2657 | 151 | 2 | 135 |  | 1.32 |
| G9-3 | 2432 | 153 | 2 | 107 |  | 1.31 |
| G9-4 | 2564 | 145 | 2 | 105 |  | 1.38 |
| Mean |  |  |  |  |  | 1.25 |
| SD |  |  |  |  |  | 0.18 |
| Mouse ID | beta cells counted | PHH3+ cells | ALDH+PHH3+ cells | ALDH+PHH3- cells | %ALDH+beta cells | %ALDH+ cells of PHH3+ cells |
| G9-1 | 2845 | 35 | 0 | 145 |  | 0.00 |
| G9-2 | 2958 | 53 | 1 | 128 |  | 1.89 |
| G9-3 | 2678 | 43 | 0 | 127 |  | 0.00 |
| G9-4 | 2824 | 38 | 0 | 122 |  | 0.00 |
| Mean |  |  |  |  |  | 0.47 |
| SD |  |  |  |  |  | 0.94 |
